# Call to Action for Enhanced Equity and Inclusion in Cannabis Research

**DOI:** 10.1089/can.2020.0149

**Published:** 2021-04-15

**Authors:** Renée Martin-Willett, L. Cinnamon Bidwell

**Affiliations:** ^1^ Department of Psychology and Neuroscience, and University of Colorado Boulder, Boulder, Colorado, USA.; ^2^ Institute of Cognitive Science, University of Colorado Boulder, Boulder, Colorado, USA.

**Keywords:** cannabis, equity, marijuana, research methods, underrepresented groups

## Abstract

**Introduction:** Policies regarding cannabis use are rapidly evolving in the United States as exemplified by the legalization of recreational use in 11 states and the District of Columbia. Previous cannabis-related laws, however, disproportionately targeted communities of color before legalization, and many argue new policies are not being developed with the input of minority stakeholders postlegalization. Given that biomedical research has also historically underrepresented communities of color, there is an obligation on the part of researchers now to actively work toward improving equity in cannabis research at a time when the field is rapidly expanding. This is particularly important for research concerning therapeutic uses of cannabis and risk liabilities.

**Objective:** This article is a call to action to improve equity and inclusion in cannabis research design and practice. Specifically, it includes three recommendations focusing on (1) inclusiveness of recruitment, (2) improve demographic reporting in articles, and (3) strengthening publication requirements.

**Conclusion:** These efforts will enhance the shared values and ethics of our field and improve the quality and validity of our research findings moving forward.

## Introduction

Cannabis has been used throughout human history as an alternative medicine,^1–3^ first cultivated in China in 4000 B.C. Later cannabis was used as a medicinal plant (2700 B.C.), eventually disseminating to India, Western Asia,^4^ the Mexican folk medicine tradition of *curanderismo*,^5,6^ and was added to the U.S. Pharmacopeia in 1850.^7,8^ However, public perception and policies swung in the opposite direction after Prohibition and cannabis was banned from nonmedical use in 1937.^9^ It was later listed as a schedule I drug in 1971^10^ resulting in the prosecution and incarceration of millions of people for cannabis use or possession and disproportionately targeting communities of color, largely related to racialized stereotypes of Latinx and black American users as “risks” to public welfare.^9,11–16^

Given that biomedical research has also historically underrepresented communities of color,^17,18^ there is an obligation to actively work toward improving equity in cannabis research at a time when the field is rapidly expanding. This article is a call to action to improve equity and inclusion in cannabis research design and practice, with pragmatic recommendations toward meeting that goal.

## Equity Challenges Facing Cannabis Research

As recently as 2018, more than 600,000 arrests were made for cannabis-related offenses, accounting for wholly 40% of all drug-related offenses that year,^19^ despite the fact that recreational cannabis use is increasingly legal across the United States. It is also well documented that previous and current cannabis laws disproportionately target communities of color.^9,11–15^ For example, systematic profiling and deportation of Hispanic community members,^13,16^ evidence that “intensity of enforcement” of cannabis-related laws is significantly related to the income level and race of an individual,^20^ and that African American men in particular are overrepresented in drug-related arrests, especially since the U.S. War on Drugs in the 1970s led to aggressive policing of black American neighborhoods.^9,21^ Importantly, these inequities persist despite the fact that black and Hispanic Americans use cannabis at roughly the same rate as whites.^22^ Even though many people with cannabis-related convictions remain incarcerated, new policies are not being developed with the input of minority stakeholders postlegalization,^23,24^ and there is evidence that the economic benefits of the so-called Green Rush are inequitably distributed.^25^ Thus, distrust and fear of prosecution may contribute to reluctance on the part of communities of color to contribute to cannabis research.

Cannabis research participants, as in other areas of biomedical research,^26^ have also historically been and continue to be predominantly male white Americans. Many cannabis research articles do not take consideration of ethnicity or gender in study designs, and often do not report ethnicity or gender in results. In a recent meta-analysis with a pooled sample of >20,000 individuals, 72% of all participants were white and 69% were male,^27^ and in a recent review of the behavioral health effects of cannabis concentrates, 71.8% of participants were white and 64.7% were male.^28^ Importantly, 47.9% of studies in the latter review did not report gender or ethnicity at all.

Ethnicity can be differentially conceptualized^29^ or can reify harmful or socially constructed categories or stereotypes,^30,31^ suggesting race, and not structural racism is at the root of health disparities,^32,33^ but it is still important to consider in research. Lack of reporting altogether may serve to situate maleness or whiteness as *de facto* default categories or standards to which all others are compared.^34^ It also absolves researchers of the responsibility to diversify their samples, impacting the validity and generalizability of studies.^35,36^ Finally, although racial categories are not biological realities as much as social constructions that facilitate exclusion, they—in context with other critical socioeconomic factors—can inform us about the ways that discrimination and prejudice can lead to disparate health outcomes.^37^

Even when researchers are interested in increasing representation, there are problems of distrust among groups underrepresented by research, justifiably due to a long history of discrimination or mistreatment from the research or medical community,^38,39^ as well as ineffective recruitment strategies^40–43^ and stigma and fear toward cannabis research in particular.^44^ In addition, researchers may neglect to consider that the goals of the community may not align with their goals, or the benefit of research to the community may not be apparent. For example, African American respondents in a telephone survey were significantly more likely than whites to believe that their physicians exposed them to extra risk and would not fully explain research participation to them.^38^ Or in listening sessions with more than 100 community members, respondents noted that researchers had low cultural competency and humility that discouraged them from participating in research.^43^ Importantly, these issues are likely exacerbated in cannabis research by a history of cannabis-related legal profiling and prosecution, which is unique from other fields.

Despite these challenges, cannabis researchers have an obligation to renew their commitment to equity in research toward improving validity, generalizability, and quality in the field as well as equity and justice in biomedical research overall. Moves toward more inclusive recruitment strategies, more consistent demographic reporting, and more stringent publication requirements are also an opportunity to propel cannabis researchers beyond other fields of biomedical research in addressing this important and long-standing problem.

## Proposed Solution #1: Inclusive Recruitment Strategies

Although the challenges of recruiting underrepresented groups in research are well documented, there is a burgeoning literature as well as action at the professional, organizational, and governmental level to help researchers improve recruitment. In particular, the field of Community Engaged Research has published extensively on pragmatic strategies to support more equitable research.^45–51^ We will describe hereunder a series of recommendations adapted for cannabis research specifically from the guiding principles of this field.^52^

The nine guiding principles of community engaged research can be summarized as (1) improving communication of research goals that are couched in an understanding of the community's values and goals, (2) proactively building relationships that are mutually respectful, and (3) respecting that research and community goals may not align.^52^ Communicating with potential community or recruitment partners about values before commencing with a study can guide researchers on what level of engagement participants may be receptive to, or even inform research design to be more reflexive to the interests of the community.^53^ In the listening session study that was previously mentioned, community members were primarily concerned with prevention of chronic disease,^43^ thus researchers working in that area or integrating prevention into their studies would likely be more positively viewed by the community. In addition, communication with key stakeholders through interviews, qualitative focus groups, or listening sessions can reveal barriers that prevent participation before a study commences. For example, if transportation to a laboratory or campus is a barrier, study designs could include funds and infrastructure for rideshares/taxis in addition to traditional research honorariums.^50^

Communication with the community before commencing recruitment not only can better prepare a study team for a successful effort, but also build mutually respectful and long-term relationships with community groups. Some examples of this approach include proactively seeking partnerships with community-serving organizations through research presentations to leadership or organizational members, or through attending organization-sponsored events to meet community members and introduce yourself and your work. This can be especially critical to cannabis research; many people may feel wary of participation due to perceptions of cannabis use or fear of prosecution. Another approach can be the development of a research participant registry to which community members can provide basic demographic information and learn about your research program without having to immediately commit to participation. The registry can then be leveraged to communicate regularly about your work (e.g., newsletters or lay-public research reports) and for study-specific recruitment. A research registry and community-based partnerships can also serve the purpose of tracking a community's changing goals and the need for long-term engagement, including feedback of research results. These mechanisms simplify the process by which the results of research can be shared with those who participated in the study, who are often motivated beyond monetary gain by interest in the topic or in a service to society.^54^

Overall, by engaging in some or all of these practices before, during, and after a study, researchers can improve the diversity, equity, and generalizability of their work, but importantly, provide acknowledgment of the community as a key partner.

## Proposed Solution #2: Consistent Demographic Reporting

There are emerging debates surrounding the role of demographics in biomedical research, and how best to report demographics in a way that is both equitable and scientifically robust. This includes questions of when to employ expansive versus targeted reporting of gender and/or sex, or including gender categories that are nonbinary,^55^ and the interpretation of race and ethnicity when ethnic categories are not discrete. Nonetheless, reporting demographics in some form continues to be preferable to not reporting at all, as demonstrated by National Institutes of Health (NIH) and other funding agency policies on this issue.^56^ Demographics help to clearly define both experimental and control groups more often than not, thus enhancing the validity of an experiment,^57,58^ while also serving to gauge progress in the field toward equity in representation. Despite the aforementioned challenges of reporting demographics, it is possible to take a pragmatic and robust approach.

First, when comparing ethnic or gender groups, researchers should explicitly state the rationale for the selected comparisons. Our recommended best practice approach is to compare all ethnic and gender groups in similar ways. For example, models can compare all groups against each other as opposed to common current practices that include comparing whites to all other groups, men to just women, or grouping nonbinary gender with women (a practice that suggests that white maleness is a condition to which all others should be compared for differences). Regardless of the approach selected, providing transparent rationales around such choices will elevate standards of inclusivity and equity in research reporting. Also, allow for “fill in” options for describing demographics, which does not limit participants to selecting only one category. Be broadly inclusive, and only limit studies to particular groups when there is a sound scientific rationale to support that approach. And finally, allow for self-assignment measures for gender, race, and ethnicity over researcher assignment whenever possible.

## Proposed Solution #3: Updating Publication Requirements

In the light of recent global activism in support of racial justice, many scientific journals and institutional departments have come forward with statements on equity and justice in research and academia. However, many organizations face criticism for positions that are antiracist in image only, without accompanying action to support their positions. Scholarly journals can actionize their statements, however, by undertaking an evidence-based needs assessment of their publication requirements by the editorial staff, external interlocutors, or working groups to realign policies toward serving equity and inclusion in research and implementing simple guidelines toward meeting that goal.

For example, journals in the biomedical sciences are increasingly updating their publication guidelines to include new requirements such as including data files with article submissions, adherence to a particular reporting standard such as Consolidated Standards of Reporting Trials (CONSORT), or the names of colleagues who informally reviewed an article before submission. These efforts are meant to increase reproducibility and transparency in science, but they can also serve to increase equity when combined with other initiatives that are specific to this goal, such as having minimum requirements for demographic reporting, disallowing single race or single gender studies unless there is a sound scientific rationale for the exclusion of others, or flexibility in how racial and ethnic categories are reported or represented as long as it is in support of more equitable opportunities for self-identification among research participants.

## Conclusion

There is a long history of cannabis-related laws disproportionately targeting communities of color before legalization, as well as a negligence on the part of the research community to equitably include diverse groups in cannabis research. Although the recommendations listed in this study are by no means meant to be an exhaustive answer to these inequities, they do represent feasible and implementable strategies to improve research recruitment, publication practices, and journal requirements. A shared commitment from the cannabis research community to improving engagement among diverse shareholders will benefit the field.

## Abbreviation Used

CONSORT: Consolidated Standards of Reporting Trials
